# Exploring the pathogenesis of depression and potential antidepressants through the integration of reverse network pharmacology, molecular docking, and molecular dynamics

**DOI:** 10.1097/MD.0000000000035793

**Published:** 2023-11-03

**Authors:** Zhongwen Lu, Fei Gao, Fei Teng, Xuanhe Tian, Haowei Guan, Jiawen Li, Xianshuai Wang, Jing Liang, Qiangyuan Tian, Jin Wang

**Affiliations:** a College of Traditional Chinese Medicine, Shandong University of Traditional Chinese Medicine, Jinan, China; b Department of Brain Disease, Linyi Traditional Chinese Medicine Hospital Affiliated to Shandong University of Chinese Medicine, Linyi, China; c College of Traditional Chinese Medicine, Shandong University of Traditional Chinese Medicine, Jinan, China.

**Keywords:** depression, molecular docking, molecular dynamics, reverse network pharmacology

## Abstract

Depression is characterized by a significant and persistent decline in mood and is currently a major threat to physical and mental health. Traditional Chinese medicine can effectively treat depression with few adverse effects. Therefore, this study aimed to examine the use of reverse network pharmacology and computer simulations to identify effective ingredients and herbs for treating depression. Differentially expressed genes associated with depression were obtained from the Gene Expression Omnibus database, after which enrichment analyses were performed. A protein-protein interaction network was constructed using the STRING database to screen core targets. The Traditional Chinese Medicine Systems Pharmacology Database and Analysis Platform database was used to screen ingredients related to these core targets, and the core ingredients were screened by constructing the “Targets-Ingredients-Herbs” network. Drug evaluation analysis was performed using the SwissADME and ADMETlab platforms, according to Lipinski Rule of 5. The binding between the targets and ingredients was simulated using molecular docking software. The binding stability was determined using molecular dynamics analysis. The “Ingredients-Herbs” network was constructed, and we annotated it for its characteristics and meridians. Finally, the selected herbs were classified to determine the formulation for treating depression in traditional Chinese medicine. The pathogenesis of depression was associated with changes in SPP1, Plasminogen activator inhibitor 1, CCNB1 protein, CCL3, and other genes. Computer simulations have verified the use of quercetin, luteolin, apigenin, and other ingredients as drugs for treating depression. Most of the top 10 herbs containing these ingredients were attributed to the liver meridian, and their taste was symplectic. *Perilla Frutescen, Cyperi Rhizoma*, and *Linderae Radix*, the main components of “Tianxiang Zhengqi Powder,” can treat depression owing to Qi stagnation. Epimedium and Citicola, the main traditional Chinese herbs in “Wenshen Yiqi Decoction,” have a positive effect on depression of the Yang asthenia type. *Fructus Ligustri Lucidi* and *Ecliptae Herba* are from the classic prescription “Erzhi Pills” and can treat depression of the Yin deficiency type. This study identified the key targets and effective medicinal herbs for treating depression. It provides herbal blend references for treating different types of depression according to the theory of traditional Chinese medicine.

## 1. Introduction

Depression is a relatively common psychological illness that is associated with changes in cognition and behaviors; moreover, some patients even have negative ideas and behaviors such as self-harm and suicide.^[1]^ The COVID-19 pandemic has increased the prevalence of mental illness worldwide over the last 3 years.^[2]^ As one of the most common mental diseases, depression significantly impacts individual health and social development.^[3]^ Therefore, it is essential to investigate suitable treatment options for depression.

The global prevalence of depression has not decreased since 1990, despite numerous interventions that have reduced some of its effects.^[4]^ Studies have shown that because the complexity of depression is determined by genetic and environmental factors, the precise neural mechanism underlying depression remains unknown. Existing theories related to depression mainly include the monoamine neurotransmitter depletion, neuroplasticity, hypothalamic-pituitary-adrenal axis, and microglia activation hypotheses.^[5–8]^

Currently, drugs such as escitalopram, nortriptyline, and mirtazapine are commonly used in treating depression.^[9]^ Although these drugs have a certain therapeutic effect on depression, they have various side effects,^[10–12]^ and tolerance can easily develop if they are administered for a long time.^[13]^ Studies have shown that traditional Chinese medicine can be used to treat depression.^[14]^ Moreover, in some countries Chinese herbs are used to treat various mental diseases.^[15]^ Experiments have confirmed that Chinese herbal medicines, such as saffron, chamomile, lavender flower, and bupleurum have similar efficacy but fewer adverse reactions compared to traditional medicines.^[16,17]^ Therefore, it is desirable to obtain more effective antidepressants from Chinese herbal medicines that are better tolerated and have fewer adverse effects.

The cycle and costs of screening and developing drugs for the treatment of depression is relatively high.^[18]^ However, extracting crucial ingredients from natural Chinese herbs to treat depression could significantly reduce costs. The Traditional Chinese Medicine systems pharmacology database and analysis platform (TCMSP, https://tcmspw.com/tcmsp.php) (accessed on January 7, 2023) contains a vast amount of information on traditional Chinese medicine, diseases, and targets and provides a reliable basis for studying the treatment of diseases using Chinese herbs.^[19]^ Network pharmacology emerged in this context, and reveals the mechanism of Chinese herbal medicine in treating diseases and saves significant costs for the extraction, research, and development of drugs.^[20]^ Therefore, the practicality of investigating the disease’s critical targets in order to identify therapeutic drugs that specifically target these targets and ultimately cure the disease cannot be overstated. This approach holds the potential to enhance the accuracy of predicting therapeutic drugs and reduce the overall cost of experimental procedures. By employing the reverse network pharmacy method, a number of critical Chinese herbs have been discovered for the treatment of obesity, including *Aloe, Portulacae Herba, Mori Follum, Silybum Marianum, Phyllanthi Fructus, Pollen Typhae, Ginkgo Semen, Leonuri Herba, Eriobotryae Folium*, and *Litseae Fructus*.^[21]^ Pharmaceutical agents such as Fostamatinib, Icatibant, and Mecasermin have been recognized as indispensable therapeutic interventions for vascular dementia, employing the reverse pharmacology approach.^[22]^ This methodology not only offers novel research avenues for the exploration of therapeutic agents but also serves as the basis for the present study, which employs a similar approach to identify potential drugs for depression treatment.

**Figure 1.**
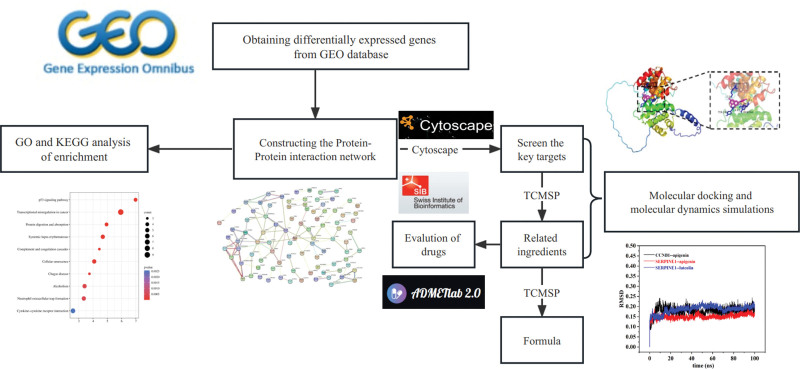
Technology roadmap for this study.

In this study, we used a novel approach to explore the treatment of depression. We used gene expression omnibus (GEO, https://www.ncbi.nlm.nih.gov/geo/) (accessed on January 3, 2023) and STRING databases to identify the core genes of depression and searched for chemical ingredients and Chinese herbal medicines that are related to core genes based on the TCMSP database and Analysis Platform. Through drug evaluation, molecular docking, and molecular dynamics analysis of core drugs, Chinese herbal medicines, and chemical ingredients with potential therapeutic effects against depression were screened. The technology roadmap used in this study is presented in Figure 1.

## 2. Materials and methods

### 2.1. Acquisition and standardization of the disease targets

Three gene expression profiles (GSE19738, GSE76826, and GSE182193) were screened from the GEO database (https://www.ncbi.nlm.nih.gov/geo/) (accessed on January 3, 2023) with “depression” as the keyword. After standardizing the gene sets using |log FC |> 1 and *P* values < .05 as the threshold value standard, we filtered out the differentially expressed genes (DEGs) as the key genes in depression using the ggplot2 package in R language (Version 3.6.3) to formulate the volcano plots of the DEGs. After aggregating and deleting duplicate DEGs, 323 DEGs were identified, which can be considered key genes associated with depression.

### 2.2. Gene ontology (GO) and Kyoto encyclopedia of genes and genomes (KEGG) pathway enrichment analysis of the hub genes

GO and KEGG enrichment analyses of DEGs were performed using the Matescape database (http://metascape.org/gp/index.html) (accessed on January 5, 2023). GO enrichment analysis annotated genetic information based on 3 aspects: molecular function, biological processes, and cell composition. The result of this analysis has led to the discernment of pertinent insights into bodily functions and corresponding biological mechanisms.^[23]^ The KEGG systematically analyzes gene function and genome information and integrates data from genomics, biochemistry, and systemic-functional omics, which is useful in incorporating genes and expression information into an overall network for research.^[24]^ Finally, GO and KEGG enrichment results were visualized using a bioinformatics website (www.bioinformatics.com.cn/) (accessed on January 3, 2023).

### 2.3. Constructing the protein-protein interaction (PPI) network and screening the core targets

We imported the selected DEGs into the STRING database (https://cn.string-db.org/) (accessed on January 5, 2023), set the minimum required interaction score to 0.400, and hid the free nodes. The PPI network was constructed and imported into Cytoscape (version 3.7.2, https://cytoscape.org/) software. The degree values of all nodes were obtained from topological analysis, and the key genes for depression were screened.

### 2.4. Screening of the relevant ingredients

The Gene Symbol of each of the core targets in the PPI network was converted into the corresponding protein name, and the protein name was searched in the TCMSP database (https://tcmspw.com/tcmsp.php) (accessed on January 7, 2023). To identify related ingredients, we retained all compounds with oral bioavailability (OB) ≥ 30% and drug-like properties (DL) ≥ 0.18. Considering that the parameters provided by the TCMSP platform are predicted using a computer software, some data may not be consistent with the actual parameters. Therefore, we searched the remaining components that did not meet the criteria of OB ≥ 30% and DL ≥ 0.18 in PubMed and retained all compounds reported in the literature. Finally, a total of 18 compounds were identified.

### 2.5. Construction of the “targets-ingredients-herbs” network

Based on the 18 components obtained using the above method, we identified traditional Chinese Medicine (TCM) herbs on the TCMSP website containing these components. Afterwards, we constructed the “Targets-Ingredients-Herbs” network in Cytoscape software, and the degree values of all compounds were obtained using topological analysis.

### 2.6. Drug evaluation of key ingredients in the “targets-ingredients-herbs” network

We used the SwissADME (http://www.swissadme.ch/) (accessed on January 15, 2023) and ADMETlab (https://admetmesh.scbdd.com/) (accessed on January 15, 2023) networking tools to verify drug resemblance and toxicity properties to evaluate the side effects and biological and chemical values of the 3 ingredients with the highest degree in the network.

### 2.7. Molecular docking of the core targets and the core ingredients

Three genes with high degree values were obtained from the PPI network diagram using Cytoscape software and retrieved from the TCMSP database. The 3 ingredients with high degree values were obtained from the “Targets-Ingredients-Traditional Chinese Herbs” network diagram for molecular docking. The ingredients corresponding to the core targets were downloaded from the TCMSP database and processed using AutoDock software (Version 4.2.6, https://autodock.scripps.edu/downloads/) for small-molecule hydrogenation. We obtained the PDB format files of the core targets from the AlphaFold protein structure database (https://alphafold.ebi.ac.uk/) (accessed on January 20, 2023), imported them into AutoDock, and set an appropriate Grid Box for molecular docking. Finally, the conformation with the lowest docking binding energy was taken as the final docking result and saved in pdbqt format. The docking results were visualized using PyMOL software (Version 2.6.0, https://pymol.org/2/).

### 2.8. Molecular dynamics simulation

GROMACS (version 2021.2) was used to simulate the molecular dynamics of the protein-active ingredients obtained through molecular docking. The protein topology file was generated using the AMBER99SB-ILDN force field, and the ligand topology file was generated using the ACPYPE script with the AMBER forefield. The system was neutralized using NaCl counterions. Before the molecular dynamics simulation, the complex was minimized for 1000 steps and equilibrated by running (canonical ensemble) and (constant-pressure, constant-temperature) for 100 ps. Subsequently, an molecular dynamics simulation was performed for 100 ns for each system under periodic boundary conditions at a temperature of 310 K and a pressure of 1.0 bar. Finally, the root mean square deviation (RMSD) and gyration radius curves of the core target bonds in the core ingredients were obtained to determine their stability.

### 2.9. Screening and annotation of the key herbs

After obtaining the chemical ingredients related to the core targets in the TCMSP database, we searched all the herbs containing these chemical ingredients in the TCMSP database and constructed a network of “Target-Ingredients-Herbs.” Using topological analysis, we obtained the top 10 Herbs in terms of degree values as alternatives to TCMS for treating depression. Referring to the Chinese Pharmacopoeia 2020 edition (I), we labeled the taste and Meridian Tropism of essential traditional Chinese herbs.

### 2.10. Constructing the network of the “targets-ingredients”, “target-ingredients - traditional Chinese herbs”, and “traditional Chinese herbs - tastes - meridian tropism”

We used the Cytoscape to build network diagrams of the “Target-Ingredients”, “Target-Ingredients - Traditional Chinese herbs”, and “Traditional Chinese herbs - Tastes - Meridian Tropism” to determine the connections between all the nodes mentioned above. The degree values of all nodes were obtained according to the network topology analysis. We then analyzed the characteristics and commonalities of traditional Chinese medicine in treating depression according to the degree values.

### 2.11. Classifying the selected herbs with the traditional Chinese medicine theory and literature

We classified traditional Chinese herbs with higher degree values according to the “Target-Ingredients - Traditional Chinese Herbs” network. Combined with traditional Chinese medicine literature, we connected and arranged the ingredients according to their degree values and found some formulas that used them as primary treatment herbs. Based on the classification of depression, we classified these therapeutic herbal pairs and formulas as representative of the treatment of different types of depression.

## 3. Results

### 3.1. Identification of differentially expressed genes in depression

**Figure 2.**
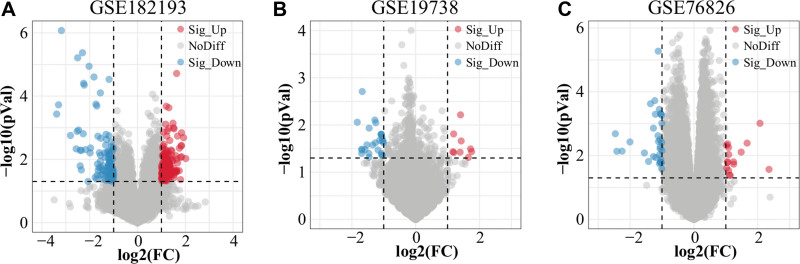
Volcano plot to identify differentially expressed genes. (A) GSE182193, (B) GSE19738, and (C) GSE76826.

We standardized the datasets and screened out 323 DEGs (| logFC | > 1, *P* < .05) from GSE19738, GSE76826, and GSE182193 after merging and removing duplicated DEGs, including 35 genes (9 upregulated genes and 26 downregulated genes) in the GSE19738 dataset, 48 genes (16 upregulated genes, 32 downregulated genes) in the GSE76826 dataset, and 245 genes (138 upregulated genes, 107 downregulated genes) in the GSE182193 dataset as shown in the volcano plot (Fig. 2). The abscissa in the volcano plot is the log2 (fold change) value, and the ordinate is the log10 (*P* value). A total of 323 DEGs were identified by combining the DEGs and removing duplicates.

### 3.2. Construction of PPI network and identification of Hub genes

**Figure 3.**
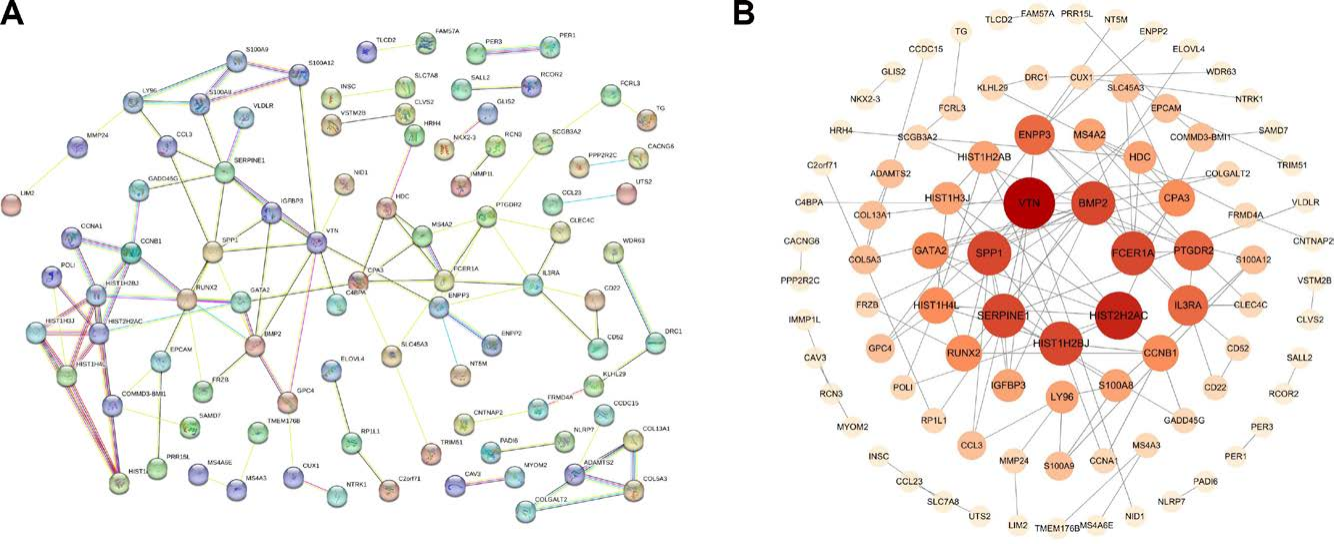
The PPI network. (A) STRING database image, (B) The Cytoscape topology analysis Network image. PPI = protein-protein interactions.

By importing 323 DEGs into the STRING database (https://cn.string-db.org/), setting the minimum required interaction score to 0.400, and hiding free nodes, we obtained a model with 90 nodes. The Network with 114 edges (Fig. 3A) was imported into Cytoscape software (version 3.7.2) to analyze the degree values of all nodes. We sorted the size of all nodes according to their degree values, such that genes with higher degree values had larger nodes and darker colors in the graph (Fig. 3B). Among them, the degree values of VTN, SPP1, plasminogen activator inhibitor 1 (SERPINE1), membrane spanning 4-domains a2, HIST2H2AC, and other genes were high in the whole network; therefore, these genes can be considered crucial for the development of depression.

### 3.3. GO and KEGG enrichment analysis of hub genes

**Figure 4.**
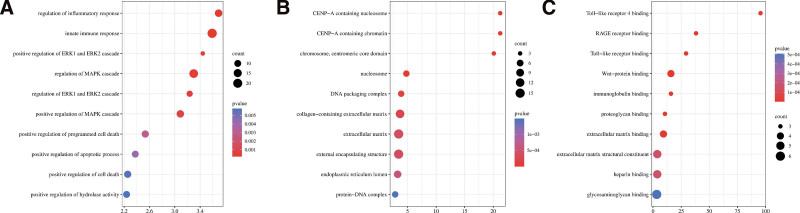
GO enrichment analysis. (A) BP based on GO analysis of depression-related hub genes, (B) CC based on GO analysis of depression-related hub genes, and (C) MF based on GO analysis of depression-related hub genes. The X-axis label represents the values of count and the Y-axis label represents the pathway. BP = biological processes, CC = cellular components, GO = gene ontology, MF = molecular functions.

Bubble charts were mapped using the bioinformatics website (http://www.bioinformatics.com.cn/). As shown in Figure 4, hub genes were significantly enriched in the regulation of inflammatory response, positive regulation of ERK1 and ERK2 cascade, regulation of ERK1 and ERK2 cascade, positive regulation of programmed cell death, positive regulation of hydrolase activity for biological processes, CENP-A-containing nucleosome, chromosome, centromeric core domain, DNA packaging complex, extracellular matrix for cellular components, and Toll-like receptor 4 binding, Wnt-protein binding, and proteoglycan binding for molecular functions.

**Figure 5.**
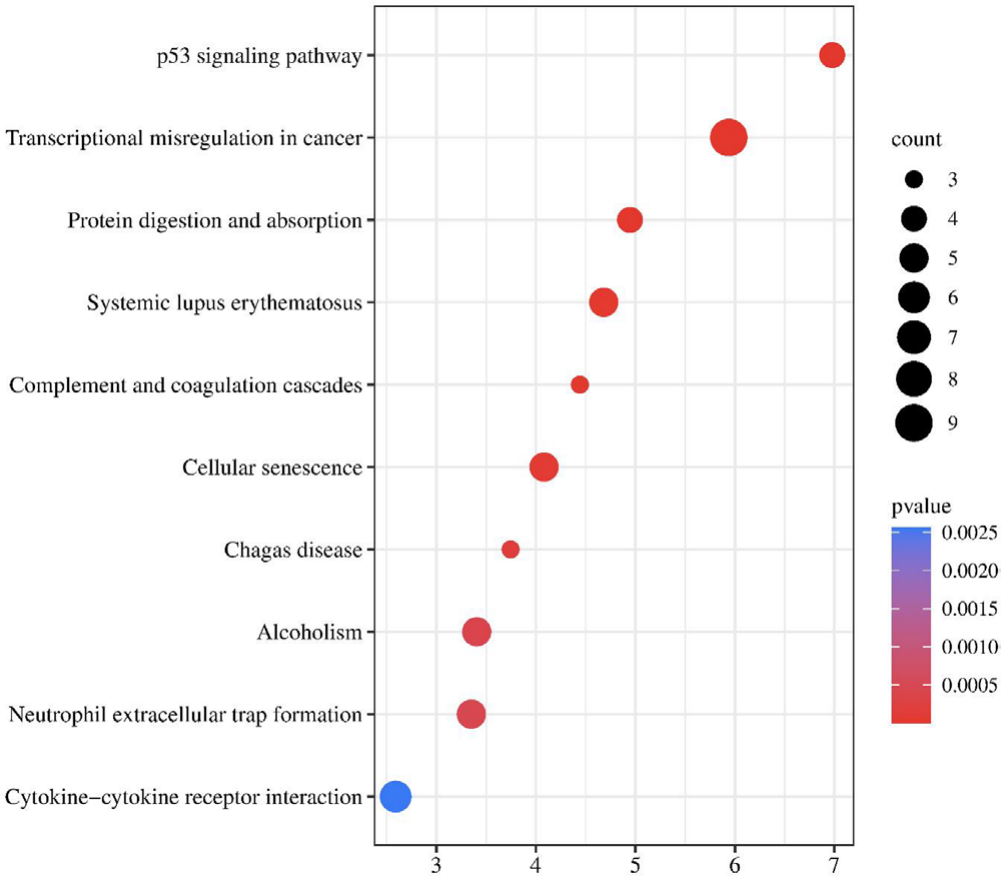
KEGG enrichment analysis. KEGG pathway analysis of hub genes. The X-axis label represents the values of count and the Y-axis label represents the pathway. KEGG = Kyoto encyclopedia of genes and genomes.

The KEGG results showed that the hub genes were mainly enriched in the following: the p53 signaling pathway, transcriptional misregulation in cancer, protein digestion, and absorption, complement and coagulation cascades, systemic lupus erythematosus, and neutrophil extracellular trap formation. The results of the KEGG pathway enrichment analyses are shown in Figure 5.

### 3.4. Screening of the relevant ingredients

We converted the 32 hub genes with a degree greater than or equal to 3 in the PPI network into protein names in the Universal Protein Resource database (https://www.uniprot.org/). These protein names were searched in the TCMSP database (https://old.tcmsp-e.com/tcmsp.php) to identify potential herbal medicines with therapeutic effects against depression. After excluding genes that were not present in the TCMSP database, we obtained 10 core genes that correspond to a total of 35 chemical ingredients. We screened all the chemical ingredients with OB ≥ 30% and DL ≥ 0.18, and combined them with the chemical members with clear experimental validation or clinical reports to prove their antidepressant effects in the PubMed database. Overall, a total of 18 chemical constituents were obtained, including ellipticine, luteolin, aloe-emodin, baicalein, oroxylin a, quercetin, chelidonine, genistein, cucurbitacin b, eupatilin, resveratrol, beta-elemene, lupeol, apigenin, progesterone, rosmarinic acid, chrysin, and (−)-epigallocatechin-3-gallate.

### 3.5. Construction of the “targets-ingredients-herbs” network

**Figure 6.**
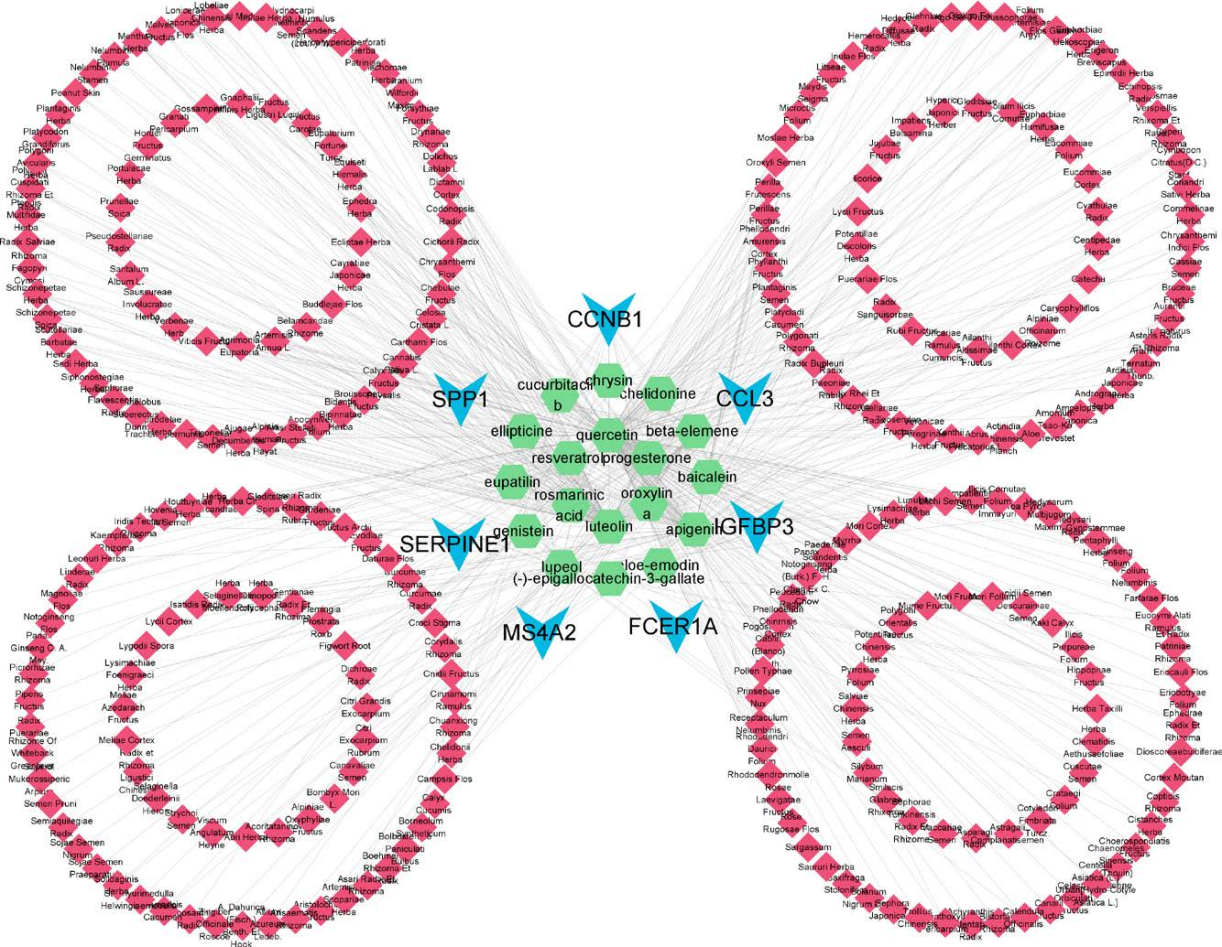
Network of “Targets-Ingredients-Herbs”.

The herbs containing these ingredients were found on the TCMSP website, and the “Targets-Ingredients-Herbs” network with 315 nodes and 539 edges was constructed in the Cytoscape software, as shown in Figure 6. In the network, blue nodes represent targets, green nodes represent ingredients, and red nodes represent traditional Chinese herbs. Topological analysis was performed on the “Targets-Ingredients-Herbs” network (Fig. 6), where the degree values of each chemical component are shown in Table S1, Supplemental Digital Content, http://links.lww.com/MD/K472. Topological analysis showed that quercetin had the largest degree value; therefore, quercetin can be considered a key component in the treatment of depression.

### 3.6. Drug evaluation of crucial compounds in the “targets-ingredients-herbs” network

According to (Table S1, Supplemental Digital Content, http://links.lww.com/MD/K472), we selected 3 ingredients with the highest degree for drug evaluation. These ingredients are quercetin (degree: 191.0), luteolin (degree: 94.0), and apigenin (degree: 84.0). The drug-likeness and toxicity of quercetin, luteolin, and apigenin were evaluated based on their molecular weight (≤500), hydrogen bonding acceptor (≤10), hydrogen bonding donor (≤5), MlogP (≤4.15), bioavailability score (>0.1), and topological surface area (<140). The results showed that quercetin, luteolin, and apigenin could be used as novel drugs based on their pharmacokinetic parameters, as shown in Table 1.

**Table 1:** Physicochemical properties.

	Compound	Lipinski rules			Moriguchi octanol-Water Partition Coefficient	Lipinski Violations	Bioavailability score	Topological surfacearea
		Molecular weight	Hydrogen bonding acceptor	Hydrogen bonding donor				
1	Luteolin	286	6	4	−0.03	0	0.55	111.13
2	Apigenin	270	5	3	3.307	0	0.55	90.9
3	Quercetin	302	7	5	−0.56	0	0.55	131.36

Despite their acceptable therapeutic value, these drugs are still unavailable as final products owing to their unintended toxicity. Therefore, these drugs require further testing via computer simulations. Quercetin, luteolin, and apigenin levels were evaluated using the ADMETlab platform for HERG blockers, to determine their acute oral toxicity in rats, eye corrosion, and respiratory toxicity (including LD50 [5.238 mg/kg]), as shown in Table 2. These results suggest that these compounds can be safely used to treat depression.

**Table 2:** Toxicity profile.

Compound	Luteolin	Apigenin	Quercetin
HERG blocker	Nor-blocker	Nor-blocker	Nor-blocker
Rat oral acute toxicity	Negative	Negative	Negative
Eye corrosion	Negative	Negative	Negative
Respiratory toxicity	Negative	Negative	Negative
LD50	5.302	5.209	5.331

### 3.7. Molecular docking verification of hub genes and core ingredients

**Figure 7.**
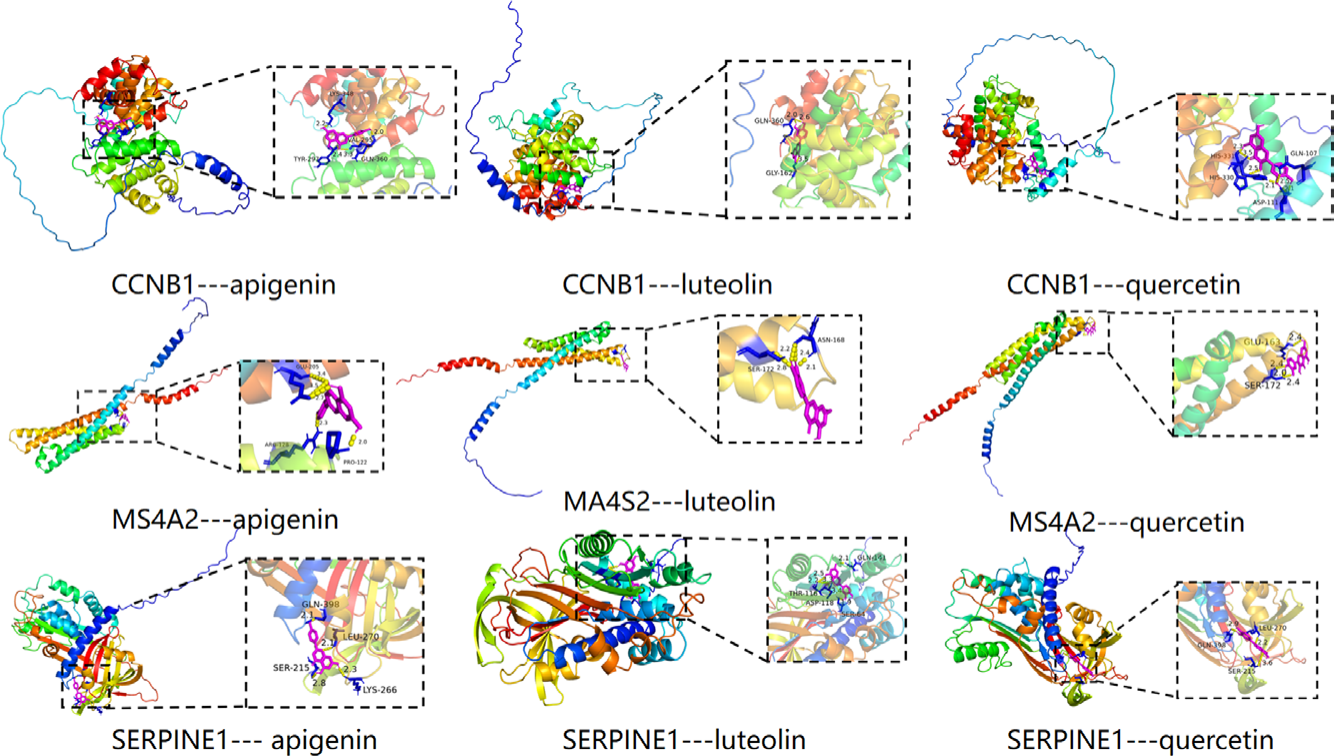
Molecular docking results for core ingredients and targets.

Three DEGs with high degree values in the PPI Network that could be searched in the TCMSP database were selected and combined with the top 3 chemical ingredients in terms of the degree values in the “Ingredients-Targets” network diagram (Fig. 6) for molecular docking. The molecular docking results are presented in Table 3. The results showed that all chemical ingredients could bind to the hub genes through stable hydrogen bonds, and their binding energies were all less than −4.0 kcal/mol. Among them, the binding energy of SERPINE1 and apigenin was −6.29 kcal/mol, which showed the best binding effect. Molecular docking results were visualized using PyMOL software (Fig. 7).

**Table 3:** Molecular docking results of core ingredients and core targets.

					Hydrogen bond interactions
Protein	UniProt ID	Ligand	PubChem ID	Binding Energy (kcal/mol)	Amino acid residue
CCNB1	A0A7L3T7Q8	Quercetin	5280343	−4.76	HIS-331, HIS-330, ASP-111, GLN-107
CCNB1	A0A7L3T7Q8	Luteolin	5280445	−5.16	GLN-360, GLY-162
CCNB1	A0A7L3T7Q8	Apigenin	5280443	−5.53	LYS-348, TYR-297, VAL-295, GLN-360
MS4A2	A0A4W2IFG0	Quercetin	5280343	−4.01	GLU-163, SER-172
MS4A2	A0A4W2IFG0	Luteolin	5280445	−4.01	ASN-168, SER-172
MS4A2	A0A4W2IFG0	Apigenin	5280443	−4.26	GLU-205, ARG-128, PRO-122
SERPINE1	A0A6J2EJY8	Quercetin	5280343	−4.65	LEU-270, GLN-398, SER-215
SERPINE1	A0A6J2EJY8	Luteolin	5280445	−5.91	THR-116, ASP-118, SER-64, GLN-141
SERPINE1	A0A6J2EJY8	Apigenin	5280443	−6.29	GLN-398, SER-215, LYS-266

CCNB1 = CCNB1 protein, MS4A2 = membrane spanning 4-domains a2, SERPINE1 = plasminogen activator inhibitor 1.

### 3.8. Simulation of molecular dynamics

**Figure 8.**
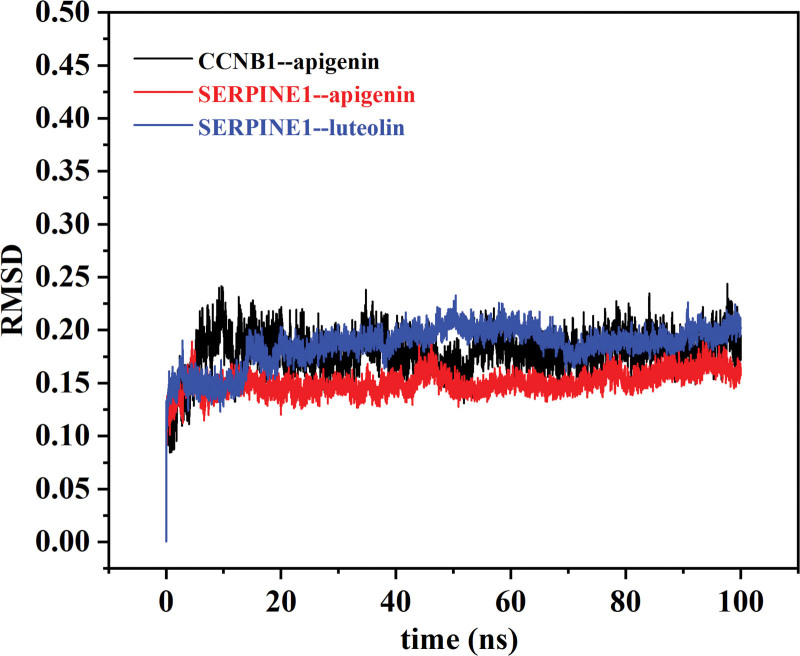
RMSD plot during molecular dynamics simulations. The X-axis label represents the time(ns) and the Y-axis label represents the root mean-square deviation. RMSD = root mean square deviation.

The RMSD curve (Fig. 8) in the molecular dynamic simulation represents the conformational fluctuations of the protein. The RMSD curve fluctuated in the early stages owing to interactions between the complex and the solvent. However, CCNB1 protein (CCNB1)--apigenin, SERPINE1--apigenin, and SERPINE1--luteolin all rose briefly before leveling off, indicating that the conformation of the protein did not change significantly after the binding of the small-molecule ligand to the protein, and the binding of the two were relatively stable.

**Figure 9.**
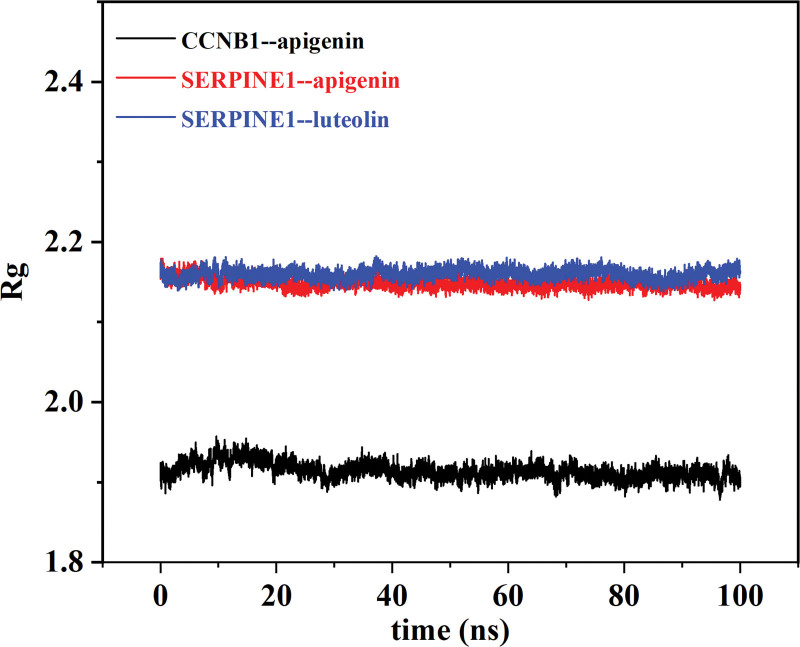
Rg plot for molecular dynamic simulations. The X-axis label represents the time(ns) and the Y-axis label represents the gyration radius.

The Rg (Fig. 9) is a curve used to describe the changes in the overall structure of a protein and can show the compactness of the comprehensive system. It can be seen from the figure that CCNB1--apigenin, SERPINE1--apigenin, and SERPINE1--luteolin all have a stable gyration radius, which is consistent with the RMSD curve, thus proving the stability of the protein conformation.

### 3.9. Screening and annotation of the core herbs

The TCMSP database contains 290 herbs with these chemical ingredients. We used Cytoscape software to build a relationship network between all the chemical ingredients in the database and their related traditional Chinese herbs. Therefore, according to the degree value in the network, we selected the top 10 Chinese herbs, including *Scutellariae Radix, Carthami Flos*, and *Chrysanthemi Flos*, which contained more chemical ingredients related to the DEGs of depression. We then annotated the 10 herbs, including their properties, tastes, and meridian tropism. (Table 4). The degree values for each herb in the network are listed in (Table S1, Supplemental Digital Content, http://links.lww.com/MD/K472).

**Table 4:** Annotation for the Chinese pharmaceutical properties of hub genes.

Herbs	Characters	Tastes	Meridian tropisms
Carthami Flos	Warm	Symplectic	Heart, liver
Menthae Herba	Cool	Symplectic	Lung, liver
Scutellariae Radix	Cold	Bitter	Lung, gallbladder, spleen, large intestine, small intestine
Gossampiniflos	Cold	Sweet, tasteless	Large intestine
Schizonepetae Spica	Warm	Symplectic	Lung, liver
Oroxyli Semen	Cool	Bitter, sweet	Lung, liver, stomach
Chrysanthemi Flos	Cold	Sweet, bitter	Lung, liver
Perilla Frutescens	Warm	Symplectic	Lung, spleen
Ephedra Herba	Warm	Symplectic, bitter	Lung, bladder
Schizonepetae Herba	Warm	Symplectic	Lung, liver

### 3.10. Construction of the “targets-ingredients- herbs - characteristics” and “herbs - tastes- meridian tropism” networks

**Figure 10.**
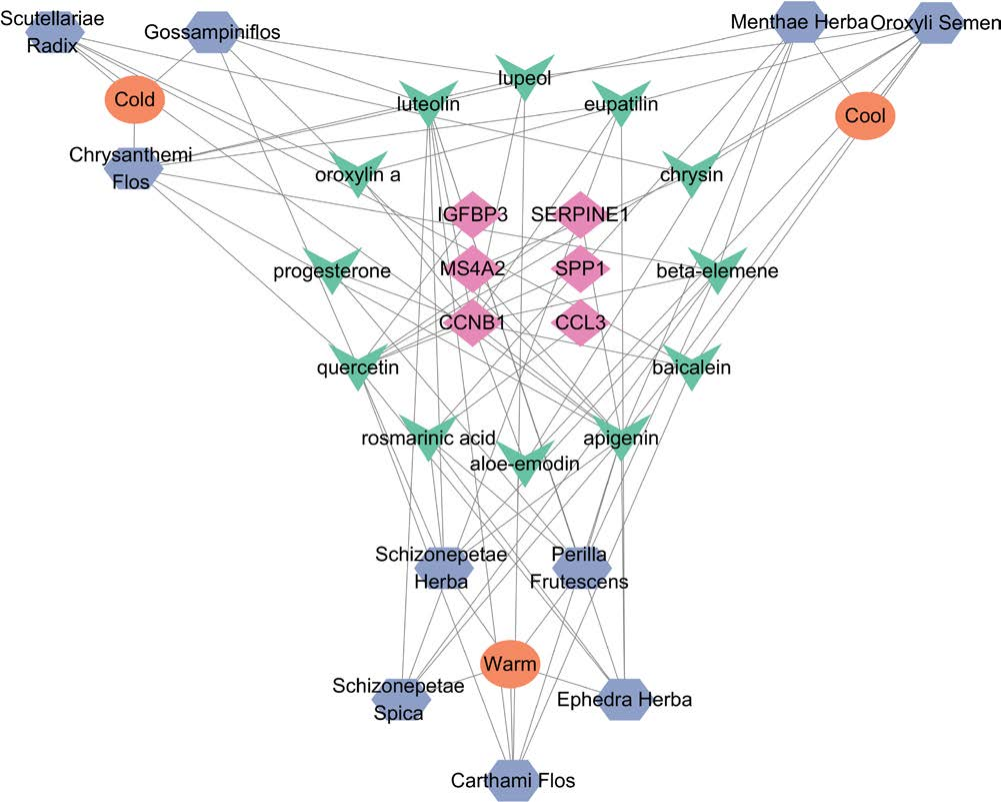
Network of “Targets-Ingredients-Herbs-Properties”.

**Figure 11.**
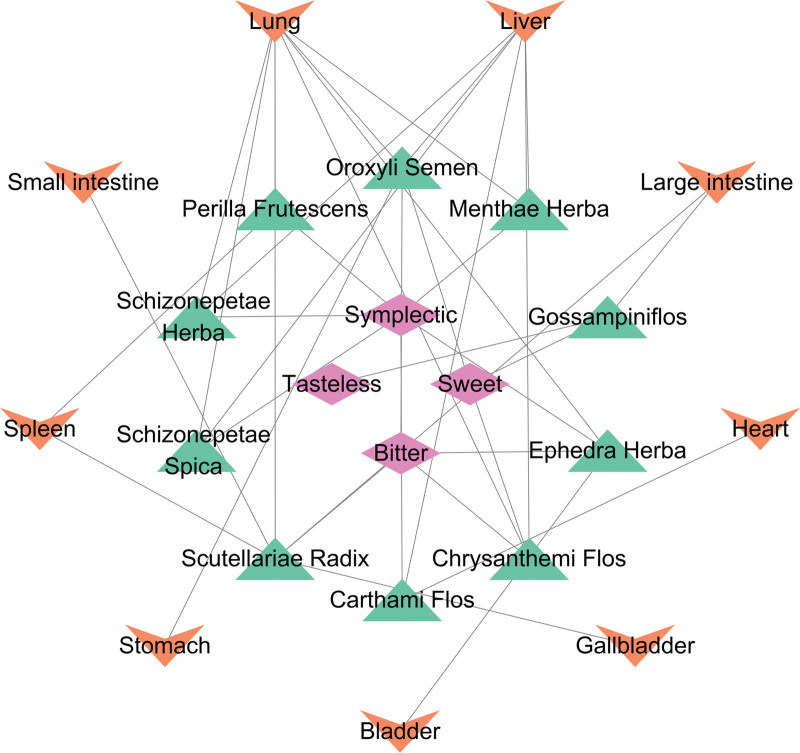
Network of “Herbs - Tastes- Meridian tropism”.

We built a network diagram of “Targets-Ingredients-Herbs-Properties” with 31 nodes and 77 edges to clarify the distribution of the herbs (Fig. 10). Six cyan nodes represent hub genes, 12 green nodes represent ingredients, 3 purple nodes represent traditional Chinese medicine properties, and 10 blue nodes represent herbal medicines. Among them were 5 warm herbs; the taste of 6 Chinese herbs was symplectic, and the 6 Chinese herbs were attributed to the liver meridian. We also drew the diagram of “Ingredients-Herbs - Meridian tropism” (Fig. 11), from which we can see that most herbs belong to the liver meridian and are warm. In traditional Chinese medicine, the liver functions to smooth emotions; therefore, drugs attributed to the liver meridian can have a positive therapeutic effect on depression. Medications with warm properties can dissipate the accumulated qi, thereby dispersing and relieving depression. Screening these drugs will provide valuable Chinese herbs and drug references for the treatment of depression.

### 3.11. Classifying the selected herbs with the traditional Chinese medicine theory and literature

Using the degree values as the criterion for screening herbs is limited, and the core point of TCM theory is syndrome differentiation and treatment. Therefore, we combined the degree values of the screened herbs and provided different treatment references for patients with varying symptoms of depression according to the theory and the characteristics of the herbs. We can roughly classify depression into Qi stagnation depression (due to impairment of Qi movement in the entire body; moreover, the absence of unimpeded Qi movement will lead to abnormal emotional responses), yang asthenia depression (weakening of Yang Qi’s promoting effect can lead to mental listlessness), and yin asthenia anxiety depression (Yin can keep people in a peaceful state; therefore, if a person becomes hyper and restless, Yin is asthenic, and this can lead to anxiety-type depression).

“Tianxiang Zhengqi Powder” (from the Subtle Meaning of the Jade Swivel) is prescribed for the treatment of Qi stagnation and delayed menstruation. Among these, *Perilla frutescens* (degree = 5), *Cyperi Rhizoma* (degree = 3), and *Linderae Radix* (degree = 2) were screened in this study. These 3 herbs play the most crucial role in the entire prescription and can regulate the stagnation of Qi. The restoration of unobstructed Qi movement resolves stagnation; moreover, as the Qi movement returns to normal, the patient’s mood improves. “Wenshen Yiqi Decoction” (from Formulation of Ziwei Zhang) uses a combination of *Epimedium* (Degree = 3) and *Citicola* (Degree = 2), which plays a vital role in the warmth of kidney Yang; therefore, it has excellent therapeutic effects on depression characterized by low mood, which is the primary symptom of Yang asthenia. If an individual’s Yang Qi is sufficient, Qi’s promotion and excitation functions are normal, and such a person would not have a low mood. *Fructus Ligustri Lucidi* (Degree = 3) and *Ecliptae Herba* (Degree = 3) form the classic prescription “Erzhi Pills” (from Subtle Formulation for Longevity). After thousands of years of testing, it has been proven that it has a good role in nourishing yin and plays a good role in patients with anxiety and depression associated with yin asthenia. Yin can restrain Yang; the body will not be agitated, and the mood will become peaceful.

## 4. Discussion

We screened depression-related DEGs in the GEO database and combined them with the TCMSP database in this study and concluded that the occurrence of depression was closely related to changes in SPP1, SERPINE1, CCNB1, CCL3, and other genes. Research has shown that the plasminogen activator inhibitor type 1 gene (SERPINE1) is associated with stress, and mutations in SERPINE1 lead to the development and progression of depression; furthermore, genetic variants of SERPINE1 may cause major depressive disorder susceptibility.^[25]^ An independent analysis of parallel response monitoring showed that SPP1 is associated with the occurrence of depression.^[26]^ According to a machine-learning study, a high level of CCL3 is an inflammatory marker of mood disorders.^[27]^

In the “Ingredients-Targets” network, we screened out the essential compounds (Luteolin, Apigenin, Quercetin) which could be considered critical ingredients for treating depression. Luteolin, a natural flavonoid, has been shown to regulate different signaling pathways involved in the pathophysiology of depression through its neurotrophic effects^[28]^ in different neuronal cell lines. Relevant studies have shown that luteolin can prevent endoplasmic reticulum stress, inhibit microglial cell activation in the brain, improve individual activity to improve depression-like behaviors^[29]^ and play antidepressive and antianxiety roles in rats with post-traumatic stress disorder.^[30]^ Apigenin can upregulate brain-derived neurotrophic factor levels in parts of the hippocampus to mediate the mechanism of depression, leading to the effects of depression.^[31]^ Quercetin could alleviate LPS-induced depression-like behavior and impairment of learning in rats; its effect may be related to the regulation of the BDF-related imbalance in the expression of copine 6 and TREM1/2 in the hippocampus and PFC.^[32]^ The results of the drug evaluation also proved that quercetin, luteolin, and apigenin could be used as drugs for the clinical treatment of depression.

Dysregulation of the innate and adaptive immune systems leads to poor prognosis in patients with depression and could also impair the efficacy of antidepressants.^[33]^ Depression is twice as common in people with type 1 or type 2 diabetes than in those without diabetes and is also associated with poorer outcomes. There is increasing evidence that the common pathogenesis of depression and type 2 diabetes mellitus may be related to excessive activation of the innate immune response and dysregulation of the hypothalamic-pituitary-adrenal axis.^[34]^ Recent studies have shown that stress can lead to changes in the extracellular matrix. Specifically, increased perineuronal net deposition has been observed in rodents exposed to chronic corticosterone or persistent social defeat stress; perineuronal net is a specific form of extracellular matrix that is mainly distributed in parvalbumin-expressing inhibitory interneurons, where it regulates neuronal excitability and brain oscillations.^[35]^ The receptor for advanced glycation end products is a pattern recognition molecule that binds oxytocin and affects emotional changes in women during the peripartum and postpartum periods.^[36]^

By constructing a “Targets-Ingredients-Herbs” network, we screened 10 relevant herbs for the treatment of depression. These drugs have not only been used in traditional prescriptions for soothing the liver and relieving depression but have also been mentioned on numerous occasions in current articles on antidepressant therapies. Among these, safflower can improve stress-induced depression syndrome in surgical or stressed rats by regulating luteinizing hormone, follicle-stimulating hormone, and monoamine levels.^[37]^ Baicalin extracted from *Scutellaria baicalensis* relieves CUMS-induced depression-like symptoms and improves neuroinflammation-induced depression-like behaviors by inhibiting toll-like receptor 4 expression through the phosphatidylinositol 3-kinase, protein kinase B (AKT), and FoxO1 pathways.^[38]^ Naringenin and apigenin are the 2 components of *Chrysanthemum morifolium*. It can exert antidepressant effects by interfering with 6 metabolic pathways: tryptophan metabolism; arginine and proline metabolism; the citrate cycle; niacin and nicotinamide metabolism; phenylalanine metabolism; and alanine, aspartate, and glutamate metabolism.^[39]^ Recent studies have shown that protocatechuic acid and luteolin-7-O-glucuronide, isolated from fermented *Perilla frutescens*, exhibit significant antidepressant-like effects. Fermented *Perilla frutescens* can correct dysfunction of the hypothalamic-pituitary-adrenal axis, and modulate brain-derived neurotrophic factor, as well as increase phosphorylation of tropomyosin receptor kinase B, extracellular regulated protein kinase, cAMP response element binding protein, and cAMP response element binding protein. It can also improve the depressive behavior of sleep deprivation-induced stress mice.^[40]^

These top 10 herbs are mainly attributed to the liver meridian, and their taste is symplectic. In traditional Chinese medicine, herbs attributed to the liver meridian play a regulatory role in liver diseases, and treat depression by soothing the liver and relieving depression. Symplectic herbs can disperse and conduct Qi and are effective in resolving symptoms of depression-related to Qi stagnation, which positively affects treatment.

For the various clinical manifestations of depression, the selection of traditional Chinese herbs should be guided by syndrome differentiation and treatment. This approach involves analyzing the diverse pathogenic factors contributing to the condition. *Pseudomonas frutescens, Cyperi rhizoma, Linderae radix*, and other traditional Chinese herbs can move Qi and relieve depression. For patients with depression of the Yang asthenia type, which is characterized by a low mood, it is necessary to warm and nourish Yang Qi; therefore, herbs such as *Epimridii Herba* and *Cistanches Herba* can be used for treatment in such cases. In traditional Chinese medicine, for Yin asthenia depression, herbs such as *Fructus Ligustri Lucidi* and *Ecliptae Herba* can be used to nourish Yin.

Experimental studies have shown that alpha-cyperone is the primary *active Cyperus rotundus* compound that enhances neuroplasticity by suppressing the NLRP3 inflammasome.^[41]^ The *Perilla frutescens–cyperi rhizoma* herb group can restore 6-hydroxytryptamine and dopaminergic synapses by regulating phenylalanine, tyrosine, and tryptophan biosynthesis, tyrosine metabolism, and tryptophan metabolism pathways through volatile oil, thus preventing depression-like behavior in ovariectomized rats.^[42]^ Various natural compounds in *Epimerdii Herba* can be used to treat depression by regulating multiple signaling pathways and targets, such as IL6, AKT1, and EGF. Icariin, the primary chemical component of *Epimerdii Herba*, has antidepressant effects as it has been shown to improve hippocampal neurogenesis in rat models.^[43,44]^

It is important to acknowledge that this study has certain limitations. While the aforementioned findings are backed by theoretical principles in traditional Chinese medicine, there remains a lack of clinical studies. Consequently, additional experimental validations are necessary to establish the effectiveness and safety of these therapies. Furthermore, the identification of hub genes may be influenced by variations in gender, age, population, platform, and other factors, as we utilized 3 distinct datasets from the GEO database. Furthermore, the parameters generated by the TCMSP platform, Autodock software, and GROMACS are computationally predicted, and the accuracy of these predictions relies on the quality of the data. Consequently, the outcomes obtained from molecular docking and molecular dynamics simulations may deviate from the actual scenario.

## 5. Conclusion

In this study, we identified 32 core genes associated with depression and analyzed their signaling pathways using the Metascape database. In addition, we screened the potential natural ingredients and Chinese herbs using the TCMSP and PubMed databases. Luteolin, quercetin, and apigenin were selected as key ingredients. *Carthami Flos, Menthae Herba, Scutellariae Radix, Gossampiniflos, Schizonepetae Spica, Oroxyli Semen, Chrysanthemi Flos, Perilla frutescens, Ephedra Herba*, and *Schizonepetae Herba* were selected as key traditional Chinese herbs. Finally, the selected herbs were classified according to classical TCM formulas, which provided a reference for treating different types of depression in TCM theory. Screening of these natural compounds and conventional Chinese herbs will provide a reference for the clinical treatment of depression. Therefore, further experimental verification is required.

## Acknowledgements

We would like to extend our most profound appreciation to edits for their patient help and Editage language service(https://app.editage.com/). Moreover, we express our heartfelt appreciation to our friends and teachers for their unwavering support and understanding throughout the research process.

## Author contributions

**Conceptualization:** Zhongwen Lu, Jin Wang.

**Data curation:** Fei Gao, Jing Liang.

**Formal analysis:** Jiawen Li.

**Investigation:** Haowei Guan.

**Project administration:** Jin Wang.

**Resources:** Qiangyuan Tian.

**Software:** Fei Teng.

**Validation:** Xuanhe Tian.

**Visualization:** Fei Gao.

**Writing – original draft:** Zhongwen Lu, Xianshuai Wang.

**Writing – review & editing:** Xianshuai Wang.

## Supplementary Material
